# Systems-epigenomics inference of transcription factor activity implicates aryl-hydrocarbon-receptor inactivation as a key event in lung cancer development

**DOI:** 10.1186/s13059-017-1366-0

**Published:** 2017-12-20

**Authors:** Yuting Chen, Martin Widschwendter, Andrew E. Teschendorff

**Affiliations:** 1 CAS Key Laboratory of Computational Biology, CAS-MPG Partner Institute for Computational Biology, 320 Yue Yang Road, Shanghai, 200031 China; 2 Department of Women’s Cancer, University College London, 74 Huntley Street, London, WC1E 6AU UK; 3 UCL Cancer Institute, University College London, Paul O’Gorman Building, 72 Huntley Street, London, WC1E 6BT UK

**Keywords:** Smoking, Cancer, EWAS, Transcription factor, Regulatory network, DNA methylation, Gene expression, Causality, AHRR

## Abstract

**Background:**

Diverse molecular alterations associated with smoking in normal and precursor lung cancer cells have been reported, yet their role in lung cancer etiology remains unclear. A prominent example is hypomethylation of the aryl hydrocarbon-receptor repressor (AHRR) locus, which is observed in blood and squamous epithelial cells of smokers, but not in lung cancer.

**Results:**

Using a novel systems-epigenomics algorithm, called SEPIRA, which leverages the power of a large RNA-sequencing expression compendium to infer regulatory activity from messenger RNA expression or DNA methylation (DNAm) profiles, we infer the landscape of binding activity of lung-specific transcription factors (TFs) in lung carcinogenesis. We show that lung-specific TFs become preferentially inactivated in lung cancer and precursor lung cancer lesions and further demonstrate that these results can be derived using only DNAm data. We identify subsets of TFs which become inactivated in precursor cells. Among these regulatory factors, we identify AHR, the aryl hydrocarbon-receptor which controls a healthy immune response in the lung epithelium and whose repressor, AHRR, has recently been implicated in smoking-mediated lung cancer. In addition, we identify FOXJ1, a TF which promotes growth of airway cilia and effective clearance of the lung airway epithelium from carcinogens.

**Conclusions:**

We identify TFs, such as AHR, which become inactivated in the earliest stages of lung cancer and which, unlike AHRR hypomethylation, are also inactivated in lung cancer itself. The novel systems-epigenomics algorithm SEPIRA will be useful to the wider epigenome-wide association study community as a means of inferring regulatory activity.

**Electronic supplementary material:**

The online version of this article (doi:10.1186/s13059-017-1366-0) contains supplementary material, which is available to authorized users.

## Background

Elucidating the mechanisms of early carcinogenesis is important, not only for improving our understanding of cancer, but also for devising and implementing risk prediction and preventive action strategies [1, 2]. To this end, many studies have begun to map molecular alterations associated with major cancer risk factors in normal or precursor cancer cells [3–9]. Smoking is of particular interest since it is a potent risk factor for many cancers, especially lung cancer.

Many previous efforts have identified molecular changes in normal or cancer cells exposed to smoke carcinogens. For instance, studies of the somatic mutation landscape of a wide range of different cancer types have unraveled a somatic mutational signature that is associated with smoking exposure [4, 10]. Other studies comparing gene expression levels in the normal lung tissue adjacent to cancer in smokers vs non-smokers have identified smoking-associated gene-expression signatures [9, 11]. Epigenome-wide association studies (EWAS) conducted in blood [8, 12–14] and buccal tissue [6] have also identified highly reproducible smoking-associated differentially methylated CpGs (smkDMCs) [15]. A recent EWAS in buccal cells, a source of tissue enriched for squamous epithelial cells, also showed how many of the smkDMCs mapping to promoters, anti-correlate with corresponding gene expression changes in the normal lung tissue of smokers [6]. More recent studies have shown that many of the top-ranked smkDMCs (e.g. this includes CpGs mapping to the aryl hydrocarbon-receptor repressor [AHRR] locus) predict the future risk of lung cancer and all-cause mortality [16–22]. Some studies have even suggested that hypomethylation at the AHRR locus (and other top-ranked smkDMCs) may be causally involved in mediating the risk of smoking on lung cancer [16]. However, the biological mechanism(s) linking hypomethylation of the AHRR and other top-ranked smkDMCs to lung cancer risk remain elusive. In fact, the AHR pathway is mostly known as a toxin-response pathway, suggesting that the DNA methylation (DNAm) changes observed at the AHRR locus may merely reflect a response to smoke toxins without necessarily being causally involved [6, 23]. Consistent with this, many of the top-ranked hypomethylated smkDMCs, including those mapping to the AHRR locus, do not exhibit hypomethylation in lung cancer [6], suggesting that cells carrying these DNAm alterations are not selected for during cancer progression. Thus, the role of the AHR*-*pathway in lung cancer etiology is unclear.

Here we decided to approach this paradox from a systems-epigenomics perspective. Instead of performing single-CpG site association analysis, as is customary in EWAS, we here aimed to derive a dynamic landscape of regulatory activity of transcription factors (TFs) in lung carcinogenesis. Our rationale to focus on TFs is threefold. First, several recent studies have shown that inactivation of tissue-specific TFs in cancer is under positive selection [24–26]. Blocks in differentiation, often mediated by inactivation of tissue-specific TFs is believed to be an early event which precedes uncontrolled cell growth [27–29]. Second, cancer risk single nucleotide polymorphisms (SNP) often map to non-coding regulatory regions, including enhancers, suggesting that the risk effect may be mediated through disruption of TF binding [30]. Third, DNAm patterns offer great promise as a means of inferring tissue-specific TFs via TF binding activity [31, 32].

In order to infer regulatory activity of TFs, we devised a novel algorithm called SEPIRA (Systems EPigenomics Inference of Regulatory Activity), which aims to infer sample-specific TF binding activity from the genome-wide expression or DNAm profile of a sample. SEPIRA leverages the power of a large RNA-sequencing (RNA-seq) expression compendium encompassing thousands of samples from many different tissue types, while adjusting for cell-type heterogeneity. Although several methods for inferring TF binding activity from gene expression data exist [33–41], SEPIRA is also able to infer regulatory activity purely from the patterns of promoter DNAm change at a key set of high-quality targets. We note that computational tools to infer regulatory activity from DNAm profiles have not been extensively applied or validated [36, 37, 40]. We posited that a powerful tool for inferring regulatory activity from DNAm profiles would be particularly valuable for identifying early causal pathways in carcinogenesis, as TF binding sites are often observed to become hypermethylated in response to a wide range of different cancer risk factors, including smoking and age, which may cause, or be a reflection of, differential binding activity [6, 31, 32, 42].

Importantly, using SEPIRA, we are here able to shed new light on the potential role of the AHR/AHRR pathway in lung cancer etiology, linking its inactivation to an altered immune response in the lung epithelium, while also identifying other regulatory pathways (e.g. FOXJ1/HIF3A) which become inactivated in smoking-associated lung cancer, in precursor lung cancer lesions, and in normal cells exposed to smoke carcinogens. Specifically, our work points towards inactivation of the AHR pathway as the more fundamental event underlying smoking-mediated lung carcinogenesis, instead of AHRR hypomethylation which is not observed in lung cancer. The unbiased discovery of the AHR pathway as well as pathways involved in hypoxia (HIF3A) and mucosa-mediated clearance of lung airways (FOXJ1), demonstrates the ability of SEPIRA to identify early and potentially causal pathways in lung cancer development. As such, SEPIRA constitutes a novel approach which opens up the inference of TF binding activity to EWAS and cancer epigenome studies.

## Results

### Overall rationale and strategy

**Fig. 1:**
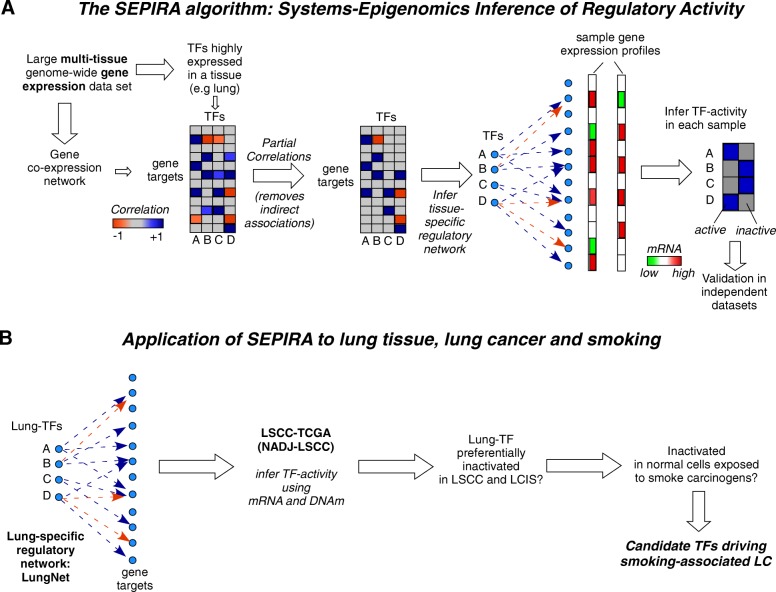
The SEPIRA algorithm and application to smoking and lung cancer. **a** The first step involves construction and validation of a tissue-specific regulatory network using the SEPIRA algorithm. This network consists of TFs significantly overexpressed in the given tissue compared to other tissue types and corresponding downstream gene targets. This network is constructed from computing co-expression correlations across a large gene expression compendium encompassing many different tissue types and subsequently using partial correlations to remove likely indirect associations. The inferred high-quality regulatory network can be used to infer TF activity in any given sample by regressing the sample’s gene expression profile against the gene target profile, encoded as 1 for activating interactions, – 1 for repression, and 0 for no significant association. SEPIRA also allows TF binding activity to be estimated from genome-wide DNAm data, regressing the gene-target promoter DNAm profile (suitably normalized, i.e. centered) of the sample against the gene-target binding profile (reversing signs relative to the gene-expression case, since lower promoter DNAm usually reflects binding activity). Finally, the tissue-specific regulatory network is validated against an independent dataset (messenger RNA expression or DNAm) encompassing many different tissue-types including the tissue-type of interest. **b** Application of SEPIRA to the case scenario of lung cancer and smoking. SEPIRA results in a lung-specific regulatory network (called LungNet, which is then used to infer TF activity in normal-adjacent (NADJ) and LSCC, as well as in lung carcinoma in situ (LCIS) (a precursor cancer lesion). This identifies TFs which become inactivated in LSCC and LCIS. A subset of these would be expected to also exhibit inactivation in the normal cell-of-origin samples exposed to the major risk factor for LSCC (i.e. smoking). We propose that inactivation of this subset of TFs could be causal mediators between smoking and LSCC

We developed SEPIRA, a novel systems-epigenomics computational method that would allow us to estimate TF binding activity in any given sample. Briefly, the algorithm begins by constructing a tissue-specific TF regulatory network consisting of: (1) TFs that are significantly more expressed in that tissue (compared to other tissues); and (2) a list of high-quality downstream gene targets (Fig. 1a). This network, as well as a regression-based method to infer TF activity from this network, is then validated in independent datasets, consisting of either gene expression or promoter DNAm patterns. Subsequently, we apply the resulting validated algorithm to the case scenario of smoking and lung squamous cell carcinoma (LSCC; a smoking-associated lung cancer), to determine whether a significant number of these lung-specific TFs become preferentially inactivated in LSCC (Fig. 1b). If true, this would indicate that their inactivation is under positive selection. Finally, we estimate TF activity in precursor lung cancer lesions and normal (epithelial) cells exposed to smoke carcinogens in order to identify a subset of the LSCC-inactivated TFs which are also inactivated in the earliest stages of carcinogenesis (Fig. 1b).

### Construction of LungNet: a lung-specific regulatory network

**Fig. 2:**
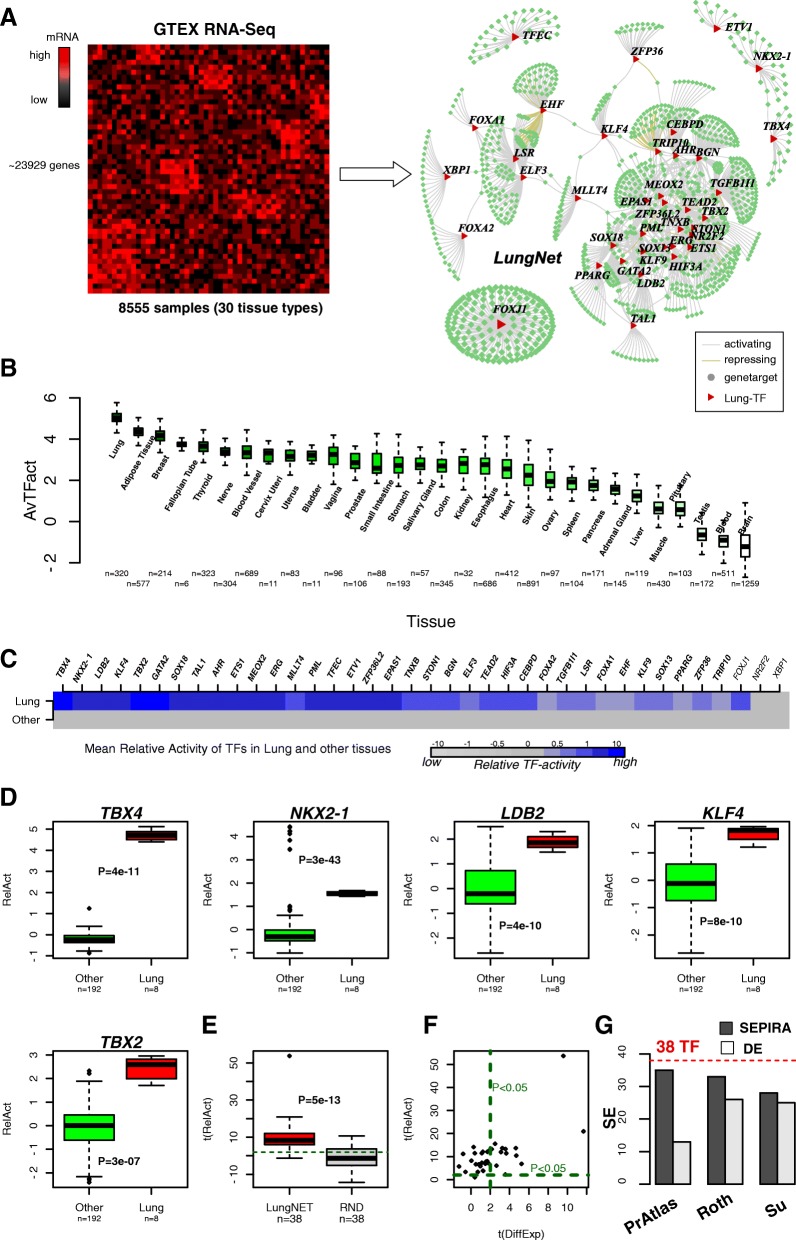
Derivation and validation of LungNet. **a** Using the multi-tissue RNA-seq compendium dataset from GTEX encompassing genome-wide gene expression measurements for > 8000 samples encompassing 30 tissue types, we inferred a lung-specific regulatory network for 38 TFs highly expressed in lung and a total of 1145 downstream gene targets. **b** *Boxplot* of TF-activity levels inferred using LungNet for each tissue-type in the same GTEX data, confirming the validity of the TF-activity estimation procedure. **c** Validation of LungNet in an independent multi-tissue RNA-seq dataset (NormalAtlas). *Color bars* compare the estimated average TF-activity levels of the 38 TFs between lung and all other 31 tissue types. In *bold*, we indicate those TFs which exhibit statistically significant higher TF-activity levels in lung. **d** Example *boxplots* of estimated TF-activity levels for five selected lung-specific TFs. *P* values are from a one-tailed Wilcoxon rank sum test. **e** *Boxplot* comparing t-statistics of differential TF activity between lung and all other tissues for the 38 TFs against the corresponding t-statistics obtained after randomizing the gene targets for each of the 38 TFs. *P* value is from a paired Wilcoxon rank sum test. **f** *Scatterplot* of t-statistics of differential TF activity (*y-axis*) against the t-statistics of differential TF expression (*x-axis*). *Green dashed lines* indicate significance threshold *P* = 0.05 for significantly positive statistics (i.e. higher activity or expression in lung tissue compared to all other tissue types). **g** Comparison of SEPIRA to simple differential expression (DE) analysis in predicting increased activity of the 38 LungNet TFs in the normal lung tissue of three independent gene expression datasets compared to other normal tissue types: the RNA-seq set from the ProteinAtlas (PrAtlas) and two microarray expression sets (Roth et al. and Su et al., see “Methods”)

Using SEPIRA, we constructed a lung-specific regulatory network (see “Methods”). The algorithm begins by identifying likely gene targets of all given human TFs by reverse-engineering a gene expression matrix into a correlation bi-partite network and subsequently using partial correlations to remove likely indirect associations [43] (Fig. 1a). We note that by estimating correlations and partial correlations over many different tissue types, that this facilitates the identification of TF-target interactions for “tissue-specific” TFs, which by definition, are active only in a relatively small subset of tissue types. In contrast, interactions of housekeeping TFs are not favored as these are active in most if not all tissues. To infer the network, we used the high-quality RNA-seq dataset from GTEX [44], encompassing expression profiles for 23929 annotated genes and 8555 samples across 30 different tissue types (see “Methods;” Fig. 2a). In the second step, the algorithm identifies TFs that are highly expressed in lung tissue relative to all other tissue types. Cell-type heterogeneity, however, can notoriously confound this task [45]. Indeed, we observed, using the ESTIMATE algorithm [46], that lung is among the epithelial tissues with the highest contamination of immune cells (Additional file 1: Figure S1). Thus, to avoid confounding by immune-cell infiltrates, lung-specific TFs were identified by first comparing lung to blood and spleen and then separately by comparing lung to all other 27 tissue types (see “Methods”). The bi-partite network was filtered to only include these lung-specific TFs and their predicted targets. This resulted in a bi-partite network of 38 TFs highly expressed in lung tissue regulating a total of 1145 gene targets (Fig. 2a), with TFs regulating on average 47 genes (number of targets was in the range of 10–152) (Additional file 2). All 38 TFs were predicted to have more positively regulated downstream targets, with many exhibiting a strong skew towards such activated targets (Additional file 1: Table S1). We refer to this resulting bi-partite TF-target network as “LungNet.”

Importantly, we point out that (not unlike other algorithms such as ARACNE [41]) the predicted targets may not be direct binding targets of the TF, but could equally well represent indirect downstream targets which faithfully measure upstream TF binding activity. To investigate this further, we used the ChIP-Atlas (http://chip-atlas.org) resource, which contains > 25,000 chromatin immunoprecipitation sequencing (ChIP-seq) profiles, including those from ENCODE and the NIH Roadmap (see “Methods”). For a total of 19 TFs in LungNet, we found corresponding ChIP-seq profiles in the ChIP-Atlas and for these we determined if there is enrichment of TF binding targets (as derived by integrating ChIP-seq binding profiles for the given TF across all available cell lines/samples in the ChIP-Atlas) among the targets inferred in LungNet. For approximately 50% of the 19 TFs (this list included AHR, CEPBD, XBP1, ELF3, PPARG, PML, ETS1, etc.) we observed significant enrichment (Benjamini–Hochberg false discovery rate < 0.05) of binding sites within ± 1 kb, 5 kb, and 10 kb of the inferred targets, as assessed using Fisher’s exact test and verified by Monte Carlo randomizations (Additional file 1: Figure S2). For > 70% of the 19 TFs, there was marginal enrichment (Fisher’s test, *P* < 0.05), further supporting the view that a substantial fraction of the inferred LungNet targets represent direct targets of the given TFs (Additional file 1: Figure S2).

Among the 38 TFs in LungNet (Table 1), many have already established roles as pro-differentiation factors in the lung epithelium. For instance, in the case of TBX2, it has been shown that in Tbx2-deficient mice differentiation of type-1 alveolar epithelial cells is compromised [47]. FOXA2 regulates airway epithelial cell differentiation and is also required for alveolarization [48, 49]. NKX2-1 is a master TF of early lung development, whereas FOXJ1 is important for the specification of the ciliated epithelium [50]. SOX13 and SOX18 are SOX TFs, which have been broadly implicated in lung morphogenesis [51]. Other TFs in LungNet, such as HIF3A, may have a distinct role: HIF3A has been shown to be highly expressed in alveolar epithelial cells and thought to be protective of hypoxic-induced damage [52]. Another example is the aryl hydrocarbon receptor (AHR), a regulator of mucosal barrier function, activation of which during lung development enhances CD4+ T-cell responses to viral infections, and which more generally may influence immune responsiveness in the lungs [53, 54]. Thus, SEPIRA has identified TFs with key roles in the establishment of a healthy lung epithelium.

**Table 1:** The 38 lung-specific TFs in LungNet and their differential activity characteristics

LungNet TF	LSCC (RNA-seq)	LSCC (DNAm)	LCIS (mRNA)	LCIS (DNAm)	Smoking (DNAm)	Smoking^a^ (mRNA-Affy)
*TFEC*	*–24.14*	18.61	2.9	5.16	*–4.58*	UP
*TBX2*	*–33.08*	*–18.69*	*–5.52*	–1.16	*–6.55*	*DN*
*FOXA2*	*–22.01*	*–17.57*	–1.43	*–7.91*	1.41	NA
*TAL1*	*–40.65*	*-18.45*	*–2.28*	–0.87	*–5.33*	*DN*
*TBX4*	*–44.91*	*–7*	*–1.68*	*–4.45*	1.61	*DN*
*NKX2-1*	*–34.8*	*–2.76*	1.73	*–2.63*	5.18	NA
*GATA2*	*–40.81*	*–11.36*	*–2.47*	–1.53	*–5.26*	NA
*EPAS1*	*–35.72*	*–32.7*	*–3.28*	*–5.25*	–1.69	*DN*
*FOXJ1*	*–9.44*	*–16.65*	*–1.73*	*–6.94*	*–1.86*	NA
*LDB2*	*–44.35*	*–11.99*	*–3.16*	2.14	*–5.32*	*DN*
*ETS1*	*–32.67*	*–5.25*	0.15	*–3.42*	*–5.37*	NA
*ETV1*	*–33.76*	*–9.3*	*–5.97*	0.25	*–2.66*	NA
*ERG*	*–33.38*	*–23.56*	*–3.16*	*–2.76*	*–5.23*	*DN*
*ELF3*	3.56	10.57	1.96	*–4.44*	5.73	NA
*SOX13*	*–30.47*	15.77	*–7.33*	–1.8	1.13	NA
*AHR*	*–21.6*	–0.61	*–2.67*	*–2.98*	1.7	NA
*PML*	*–9.33*	*–7.3*	2.82	–1.56	*–2.06*	NA
*FOXA1*	*–9.57*	11.95	–0.21	*–5.57*	5.51	NA
*MLLT4*	–1.28	*–12.25*	2.53	2.16	1.52	*DN*
*BGN*	*–9.85*	*–24.23*	*–4*	*–6.12*	3.08	NA
*ZFP36*	*–11.43*	*–7.05*	2.9	1.05	2.62	NA
*TNXB*	*–27.31*	*–12.72*	*–4.44*	*–3.58*	4.19	NA
*SOX18*	*–39.37*	*–13.51*	*–3*	1.23	*–4.88*	NA
*TEAD2*	*–19.59*	*–32.39*	*–3.26*	*–4.49*	*–2.64*	NA
*XBP1*	5.66	*–6.78*	–0.54	1.69	–0.07	NA
*MEOX2*	*–40.54*	*–3.58*	*–4.73*	–1.6	1.28	NA
*KLF4*	*–14.85*	2.89	4.05	–0.33	5.44	NA
*HIF3A*	*–36.97*	*–9.37*	*–5.85*	3.44	3.65	NA
*LSR*	9.53	4.06	2.51	*–4.1*	5.27	NA
*KLF9*	*–29.79*	*–21.26*	*–5.49*	1	*–5.1*	NA
*STON1*	*–36.61*	*–7.75*	*–4.79*	*–3.04*	1.9	NA
*PPARG*	*–25.61*	*–19.38*	–0.45	*–4.53*	*–5.77*	UP
*ZFP36L2*	*–29.34*	*–4.42*	*–3.63*	1.66	*–3.36*	NA
*CEBPD*	*–15.4*	–0.86	–0.07	–0.14	*–4.08*	NA
*TRIP10*	0.09	*–5.79*	*–3.52*	–1.22	3.94	NA
*NR2F2*	*–20.82*	*–8.14*	*–6.41*	*–7.52*	3.61	NA
*TGFB1I1*	*–15.65*	*–25.46*	*–4.86*	*–4.96*	1.52	NA
*EHF*	6.17	25.89	2.3	*–3.08*	5.98	NA

Table lists for each of the 38 lung-specific TFs, the t-statistics of differential activity in five different studies. In LSCC (RNA-seq), t-statistic reflects differential activity of LSCC relative to NADJ tissue as measured using RNA-seq of the predicted TF targets; in LSCC (DNAm) t-statistics of differential activity are shown but as inferred using promoter DNAm levels of targets, in LCIS (messenger RNA [mRNA]) and LCIS (DNAm) t-stats reflect differential activity between LCIS and NADJ tissue as assessed using gene expression and promoter DNAm levels of targets, respectively (expression and DNAm are from unmatched cases and cohorts); and finally, in Smoking (DNAm) we give the t-statistics of differential activity between buccal samples of heavy smokers compared to non-smokers (measured in smoking pack-years) and as assessed using promoter DNAm levels of targets. Numbers in italic represent cases of significant inactivation

^a^A gene expression study comparing NADJ lung tissue of smokers to non-smokers and which only provided a table of differentially expressed genes

*DN* TFs that were reported in this study to be underexpressed in the normal lung tissue of smokers, *UP* TFs reported to be overexpressed, *NA* not reported to be consistently differentially expressed across three independent studies

To verify the validity of the predicted targets in LungNet, we estimated TF activity levels in the same GTEX samples by regressing the expression profile of each sample against the predicted TF gene target profile (see “Methods”). As required, the estimated TF activity level was higher in lung tissue compared to all other tissue types for effectively all 38 TFs (Additional file 1: Figure S3), with the average TF activity highest in lung tissue (Fig. 2b). Importantly, we note that activity of these TFs was low in blood and spleen, thus confirming that their high activity in lung is driven by cells other than immune-cell infiltrates. Confirming this further, Gene Set Enrichment Analysis (GSEA) over the 1145 targets was characterized by the absence of genes marking immune-cell types (Additional file 3).

### Validation of LungNet in independent RNA-seq data

Next, we sought to validate the regulatory interactions in LungNet using independent RNA-seq data. To this end, we estimated TF activity levels for the 38 TFs in each of 200 samples, encompassing 32 different tissue types, using expression data from the RNA-seq NormalAtlas, generated as part of the ProteinAtlas project [55]. We estimated the activity level of a given TF in a given sample as the t-statistic of a linear regression of the sample’s genome-wide expression profile against the predicted gene target profile (see “Methods”), a procedure previously shown to work well [34, 56–58]. Having estimated TF activity across all samples of the NormalAtlas set, we then asked how many of the 38 TFs exhibited higher activity levels in lung tissue compared to all other tissue types. Out of the 38 TFs, 35 (92%) were predicted to be more active in lung compared to other tissue types, thus validating LungNet (Fig. 2c, d). As a negative control, we randomized the gene targets among all genes (1000 distinct randomizations), keeping the number of targets per TF fixed, which resulted in most TFs not exhibiting higher activity in lung tissue (Fig. 2e, Additional file 1: Figure S4). Of note, using TF gene expression level as a surrogate for TF activity, only 13 (i.e. 34%) TFs were predicted to be more active in lung, demonstrating that improved inference of TF activity is possible by studying the patterns of differential expression of predicted TF targets (Fig. 2f, g). To substantiate this last result further, we analyzed two additional messenger RNA (mRNA) expression datasets encompassing many normal tissue types, including lung tissue [59, 60] (see “Methods”). We posited that SEPIRA would exhibit increased sensitivity to detect lung-specific TFs in these sets compared to using differential expression. Confirming this in the Roth et al. dataset [59], out of the 38 TFs in LungNet, SEPIRA predicted 33 to be more active in the lung tissue samples compared to all other tissues combined, whereas differential expression analysis only predicted 26 (Fig. 2g). Similarly, in the Su et al. dataset [60], SEPIRA correctly predicted 28 TFs to be more active in lung, whereas simple differential expression analysis did marginally worse (25 TFs) (Fig. 2g).

### Integration of LungNet with differential DNAm patterns to predict TF activity

**Fig. 3:**
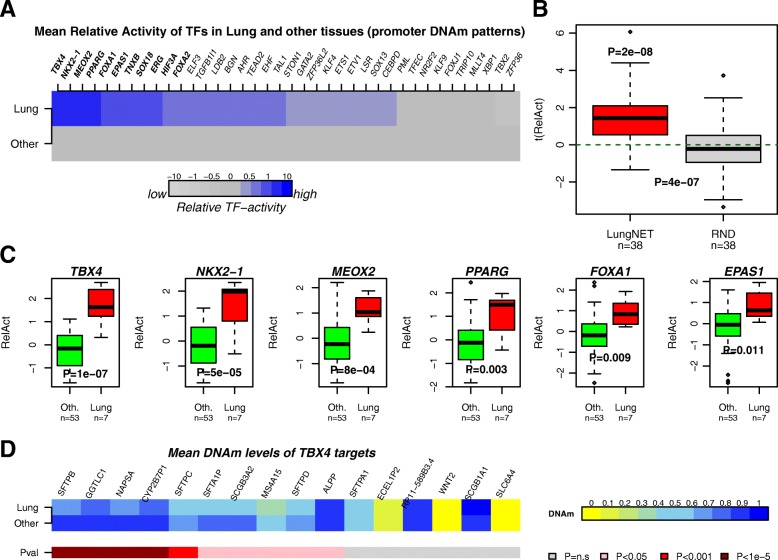
Integration of LungNet with promoter DNAm patterns. **a** *Color bars* indicate the mean relative TF activity for the 38 lung-specific TFs as estimated in the Illumina 450 k DNAm dataset for lung tissue and all other tissues combined. TFs have been sorted in decreasing order of significance with those in *bold* attaining statistical significance (*P* < 0.05). **b** *Boxplot* of t-statistics of differential TF-activity between lung and all other tissues for the 38 lung-specific TFs against the corresponding statistics for the case where the targets in LungNet were randomized. *P* values are from Wilcoxon rank sum tests. **c** *Boxplots* of estimated relative TF-activity levels between lung and all other tissues for the six top-ranked TFs. **d** *Color bars* comparing the promoter DNAm levels of the 16 TBX4 targets between lung and all other tissues, with t-test *P* values shown in *lower color bar*

Having validated LungNet, we next asked if promoter DNAm patterns at the predicted targets would also allow us to infer TF activity. This is important, as it would provide a means to infer TF activity in EWAS for which matched gene expression data are not available. We obtained Illumina 450 k DNAm data for 60 somatic tissue samples from the Stem-Cell Matrix Compendium (SCM2) [61], encompassing 11 different tissue types and including seven samples from lung tissue (see “Methods”). In order to assign a DNAm value to a gene, we used a previously validated procedure which assigns to each gene the average DNAm of probes around the transcription start site (TSS), or the average of probes mapping to the first exon if probes mapping to within 200 bp of the TSS are not available [37] (see “Methods”). Thus, we inferred activity for each of the 38 TFs in each of the 60 samples by regressing the sample’s promoter DNAm profile (centered across samples) to the corresponding gene target profile, reversing the sign of activating, and repressing interactions since low promoter methylation normally implies higher binding activity (see “Methods”). Despite the relatively small sample size (comparing seven lung vs 53 other tissues), 34 of the 38 TFs exhibited higher activity levels in lung with 11 of these 34 (FOXA2, TBX4, NKX2-1, EPAS1, ERG, FOXA1, TNXB, SOX18, MEOX2, HIF3A, and PPARG) being statistically significant (Wilcox rank sum test, *P* = 2e-8, Fig. 3a–c). We note that these results could not have been inferred using differential promoter DNAm levels of the TFs themselves (Additional file 1: Figure S5). To further check the statistical and biological significance of our result, we randomized the targets in LungNet (1000 distinct randomizations), keeping the number of targets per TF fixed, which resulted in similar numbers of positive and negative differential activity levels, with corresponding t-statistics indistinguishable from zero (Fig. 3b, Additional file 1: Figure S6). We confirmed that the higher predicted activity in lung was driven by loss of DNAm at the promoters of the predicted targets (Fig. 3d).

### Lung-specific TFs exhibit preferential inactivation in lung squamous cell carcinoma

**Fig. 4:**
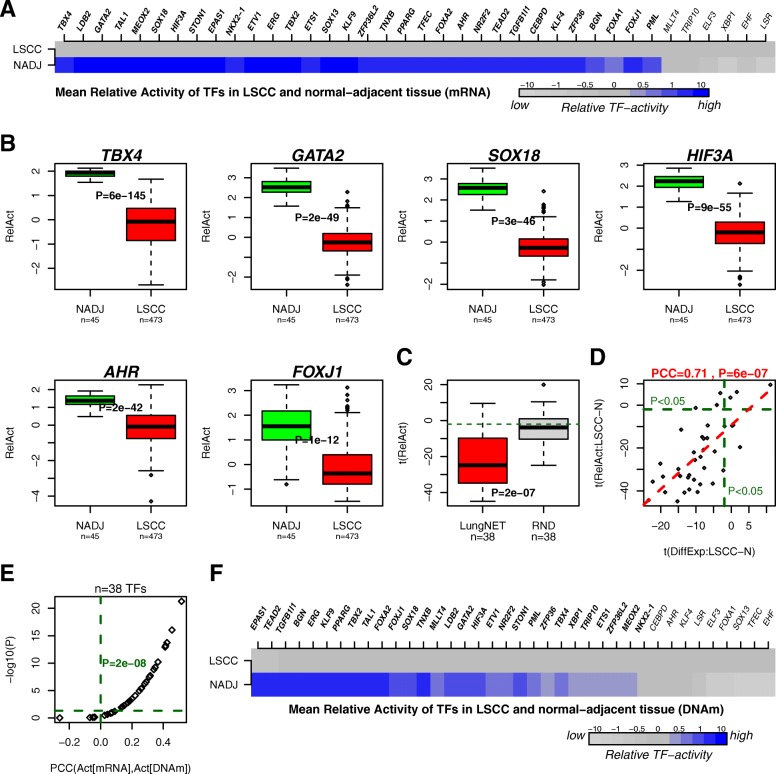
LungNet predicts preferential inactivation of lung-specific TFs in LSCC. **a** *Color bars* compare the estimated average TF-activity levels of the 38 TFs in LSCC compared to their NADJ tissue. In *bold*, we indicate those TFs which exhibit statistically significant lower TF-activity levels in LSCC. **b** Example *boxplots* of estimated TF-activity levels for six selected lung-specific TFs. *P* values are from a one-tailed Wilcoxon rank sum test. **c** *Boxplot* comparing t-statistics of differential TF activity between LSCC and NADJ for the 38 TFs against the corresponding t-statistics obtained after randomizing the gene targets for each of the 38 TFs. *P* value is from a paired Wilcoxon rank sum test. **d** *Scatterplot* of the t-statistics of differential TF activity (*y-axis*) against the t-statistics of differential expression between LSCC and NADJ tissue. *Green dashed lines* indicate line of statistical significance, with *red line* indicating the regression of y-values against x-values. Above the plot, we show the PCC and *P* value. **e** *Scatterplot* of the Pcc between the TF-activity level estimated using mRNA expression and the corresponding one estimated using DNAm (*x-axis*), against the corresponding *P* value in a –log_10_ basis (*y-axis*), for each of the 38 TFs. *Green dashed horizontal* and *vertical lines* indicate significance threshold *P* = 0.05 and PCC = 0, respectively. *P* value is from a one-tailed Wilcoxon rank sum test, testing the null hypothesis that the PCC values are drawn from a distribution centered at PCC = 0. **f** *Color bars* comparing the mean relative TF-activity levels between LSCC and NADJ, as estimated from promoter DNAm levels. In *bold*, we indicate those TFs which passed a statistical significance *P* value threshold of 0.05

Next, we inferred activity levels for the 38 TFs in the NADJ and LSCC samples from the TCGA project for which both RNA-seq and Illumina 450 k DNAm data are available (45 NADJ and 473 cancers [RNA-seq] and 41 NADJ samples and 275 cancers [DNAm]) [62]. We posited that the 38 lung-specific TFs would exhibit preferential inactivation in lung cancer, which would further support results obtained by us previously [24]. Using RNA-seq data, 32 of the 38 TFs (i.e. 84%) were significantly inactivated in LSCC (Fig. 4a, b, Table 1). To demonstrate that this result is indeed driven by LungNet, we randomized for each TF the gene targets among all available genes (keeping the number of targets per TF fixed), which resulted in only a much smaller fraction of inactivated TFs (Fig. 4c, Additional file 1: Figure S7). Of the 38 TFs, 31 were also downregulated in LSCC and we observed a strong correlation between differential TF expression and their estimated differential activity (as predicted from their gene targets) (Pearson correlation coefficient [PCC] = 0.71, *P* < 1e-6, Fig. 4d). Using the matched DNAm data, we obtained an independent set of TF-activity levels, which were in remarkably good agreement with those estimated using gene expression, with PCC values between the two sets of estimates being significantly positive (*P* < 1e-7, Fig. 4e). For 29 of the 38 TFs (i.e. 76%), their activity levels were significantly lower in LSCC as estimated using promoter DNAm levels (Fig. 4f, Table 1). Between the 32 and 29 TFs predicted to be inactivated in LSCC based on differential expression and differential methylation of their targets, respectively, we observed a strong overlap of 26 TFs, which included TBX2, FOXA2, FOXJ1, BGN, TGFB1I1, HIF3A, and SOX18 (Table 1). Finally, we verified that the inactivation of lung-specific TFs in LSCC was also seen in lung adenoma carcinoma (LUAD) (*P* = 8e-7, Additional file 1: Figure S8) and that the inactivation was significantly more pronounced in lung cancers compared to other cancer types (Additional file 1: Figure S8).

### LungNet predicts preferential inactivation of lung-specific TFs in lung carcinoma in situ (LCIS)

**Fig. 5:**
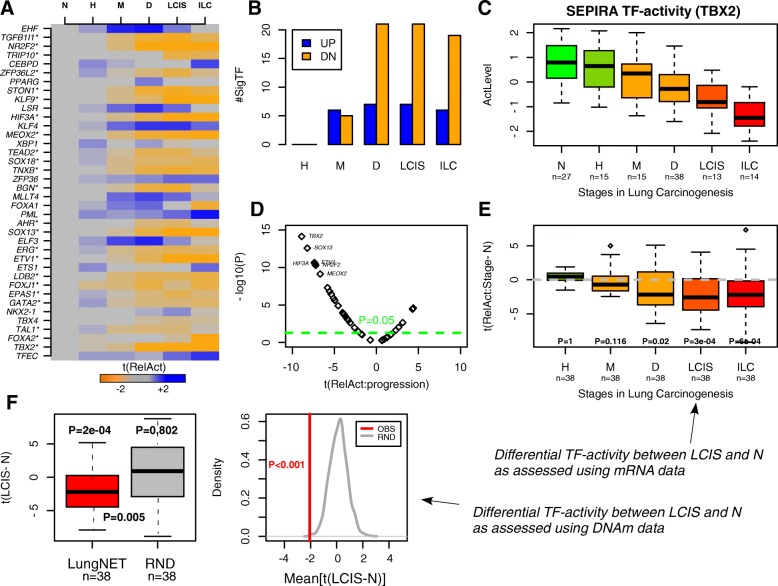
SEPIRA and LungNet predict preferential inactivation of lung-specific TFs during progression to LSCC, including LCIS. **a**–**e** RNA expression. **a** *Heatmap* of t-statistics of differential TF activity, as estimated using SEPIRA from a gene expression data matrix encompassing all major histological stages of lung carcinogenesis. *N* normal, *H* hyperplasia, *M* metaplasia, *D* dysplasia, *LCIS* lung carcinoma in situ, *ILC* invasive lung cancer (squamous). *TFs with significant changes in TF activity during disease progression. **b** Numbers of significantly deactivated (DN) and activated (UP) TFs in each disease stage relative to normal. **c** *Boxplots* of the t-statistics of differential activity between each disease stage and normal lung. *P* values are from a one-tailed Wilcoxon rank sum test, testing that the distribution of the differential activity values is < 0. **c** *Scatterplot* of t-statistics from a regression of TF-activity against disease stage (*x-axis*) against their significance level (-log_10_P, *y-axis*). **d** *Boxplot* of estimated TF-activity levels for TBX2 against disease stage. **f** DNAm. *Left*: *Boxplots* comparing the t-statistics of differential activity, estimating using SEPIRA on Illumina 450 k DNAm data, between 35 LCIS and 21 NADJ samples for the 38 LungNet TFs against a null model in which the targets of the 38 TFs were randomized among all possible targets (keeping the number of targets per TF fixed). *P* values above boxes represent the Wilcoxon rank sum test *P* values testing that the distribution of t-statistics is < 0. *P* value in-between boxes compares the distribution of t-statistics. *Right*: Density distribution of average t-statistics of differential activity obtained by performing 1000 randomizations of the targets (*gray curve*) against the observed average t-statistic of differential activity (*red vertical line*). None of the 1000 randomizations led to an average statistic lower than the observed (*P* < 0.001)

Next, we explored if the 38 lung-specific TFs also exhibit preferential inactivation in precursor lung cancer lesions, such as LCIS. We first obtained TF-activity levels in 122 lung tissue biopsies from 77 individuals, for which mRNA expression data were available, encompassing all major histological stages in the development of LSCC, including normal, hyperplasia, metaplasia, dysplasia, LCIS, and LSCC/ILC [63] (see “Methods”). From these activity levels, we computed t-statistics of differential activity between each disease stage and the normal reference (Fig. 5a). We observed a striking increase in the number of significantly inactivated TFs between the metaplasia and dysplasia stages, with the number of inactivated TFs remaining constant between dysplasia, LCIS, and invasive lung cancer (ILC/LSCC) (Fig. 5b). A formal comparison of the statistics of differential activity confirmed that the most significant inactivation occurred at the LCIS and ILC stages (Wilcoxon rank sum test, *P* < 0.001, Fig. 5c). For LCIS, 21 of the 38 TFs (i.e. 55%) were inactivated compared to the normal reference (Table 1). Using linear regressions of predicted TF activity against disease stage also revealed a clear skew towards TFs becoming inactivated, with 23 out of the 38 TFs being statistically significant (Fig. 5d) and with a subset of these (e.g. TBX2, SOX13, HIF3A) exhibiting a clear linear pattern (Fig. 5e). All these results were robust if the multiple biopsies from the same patient and disease stage were averaged before estimating TF activity (Additional file 1: Figure S9; see “Methods”). We note that, had we used gene expression levels as a surrogate for TF activity, we would have found 20 TFs to exhibit a significant linear decrease in activity with 16 specifically inactivated in LCIS, compared to the 23 and 21 TFs inferred using SEPIRA, respectively.

Next, we explored if the same pattern of preferential inactivation is also evident from analysis of DNAm data. To this end, we estimated TF-activity levels in 35 LCIS samples plus 21 NADJ lung specimens for which Illumina 450 k DNAm profiles had been generated [6]. A total of 19 TFs (i.e. 50%) exhibited significantly lower TF-activity levels in LCIS compared to NADJ tissue (Table 1). The distribution of t-statistics of differential activity of all 38 TFs was significantly < 0 (Wilcoxon rank sum test, *P* = 0.0002, Fig. 5f), further supporting the view that the TFs are preferentially inactivated. Confirming the importance of LungNet, upon randomizing the gene targets of each TF (1000 Monte Carlo randomizations), differential t-statistics were significantly less negative (Fig. 5f).

### Patterns of differential activity in normal cells exposed to smoke carcinogens

It is plausible that a fraction of the 32 lung-specific TFs inactivated in LSCC, already become inactivated in normal epithelial cells exposed to smoke carcinogens. Given that the smoking exposure information of a large gene expression dataset of normal lung tissue from smokers and non-smokers is not publicly available [11], we decided to explore this question in a large EWAS conducted in buccal tissue [6], a tissue that includes squamous epithelial cells (the type of cell thought to give rise to LSCC). Using LungNet and the DNAm profiles at the promoters of the predicted targets, we estimated TF-activity levels in the buccal samples from 790 women with varying levels of lifetime smoking exposure (measured in units of smoking pack-years [SPY]) (see “Methods”). Interestingly, we observed 15 TFs which are less active in smokers (Table 1) and there was no preference for inactivation over activation (Wilcoxon rank sum test, *P* = 0.38). Among the 15 TFs were several (e.g. TBX2, TAL1, GATA2, FOXJ1, PPARG, ETS1, ERG, ETV1, TEAD2, and PML) which also exhibited inactivation in LSCC and LCIS.

We also mapped our 38 LungNet TFs onto a list of genes differentially expressed between NADJ lung tissue of smokers vs non-smokers [11]. A total of nine TFs exhibited consistent differential expression in the three independent studies considered in [11], of which, interestingly, seven exhibited underexpression in the normal lung tissue of smokers (Table 1). These seven included three (TBX2, TAL1, and ERG) which also exhibited inactivation in the buccal tissue of smokers.

## Discussion

Using a novel systems-epigenomics approach, we have derived a landscape of TF regulatory activity in lung cancer, precursor lung cancer lesions, and normal cells at risk of neoplastic transformation. Among the lung-specific TFs inactivated in lung cancer and precursor lesions, and which may be implicated in early causal pathways, it is worth highlighting the following:

1. the TF FOXJ1 was found to be inactivated in LSCC, LCIS, and marginally so in buccal tissue of smokers (Table 1). FOXJ1 is a master TF for the generation of airway epithelial ciliated cells, which play a central role in clearing the lung of inhaled pathogens and xenobiotics. Cilia length, in particular, is important for airway clearance [64] and in vivo studies have shown that the airway epithelium of smokers has shorter cilia than that of non-smokers [65], suggesting that TFs responsible for cilia growth become inactivated in smokers and that this may contribute to related pathologies such as lung cancer [66, 67]. Interestingly, a recent study has shown that components of cigarette smoke suppress genes involved in cilia growth and that by stimulating ciliogenesis via FOXJ1 overexpression, partial re-expression of cilia-growth related genes can be achieved [68]. Thus, our analysis strongly supports a model in which inactivation of FOXJ1 may contribute causally to lung cancer progression;
2. a related TF, acting upstream of FOXJ1, is FOXA2, which we observed to be also inactivated in LSCC and LCIS (Table 1). FOXA2 has established roles in lung morphogenesis, with deletion of FOXA2 leading to inhibition of lung differentiation markers, including FOXJ1 [69]. Furthermore, it has been observed that targeted disruption of Foxa2 in the mouse lung inhibited cell maturation, causing goblet cell hyperplasia in the lung airways [69]. Interestingly, the goblet cell’s role is to enable secretion of airway mucus, whose function is to protect the lung (through mucociliary clearance) against foreign particles and chemicals entering the lung [69]. Thus, FOXA2 inactivation and goblet cell dysfunction may facilitate exposure of the lungs to more harmful particles/viruses;
3. the TF AHR was found inactivated in LSCC and LCIS, although not in buccal tissue of smokers. The observed inactivation in LSCC and LCIS is of great significance given that the locus of its repressor (AHRR) is observed to be consistently and reproducibly hypomethylated in buccal, blood, and lung tissue of smokers [6, 23, 70]. The hypomethylation of the AHRR locus in normal cells exposed to smoke carcinogens is consistent with its observed overexpression in normal lung tissue of smokers [11, 23]. Here, too, we observed overexpression of AHRR in the normal lung tissue of smokers compared to ex-smokers (Additional file 1: Figure S10A) and interestingly this overexpression was also seen in hyperplasia, metaplasia, dysplasia, and even in LCIS (Additional file 1: Figure S10B). However, AHRR overexpression and hypomethylation of the AHRR locus is not observed in LSCC (see Additional file 1: Figure S10C and [6]), suggesting that AHRR overexpression merely reflects a response to smoke toxins. In contrast, the predicted loss of TF binding activity of AHR in LSCC and LCIS parallels its observed underexpression in LSCC and LCIS (Additional file 1: Figure S10E-F), while AHR underexpression or inactivation is not observed in early lesions or in normal cells exposed to smoke carcinogens (Additional file 1: Figure S10D, E, Table 1). This last observation is not inconsistent with recent reports of an increase in enhancer activity at a few AHR regulatory elements in exposed normal cells [23]. At present it is unclear why the observed overexpression of AHRR in early lesions and exposed normal cells may not result in reduced expression and binding activity of AHR. However, the relation between AHRR and AHR is complex due to a negative feedback loop, with AHR acting to overexpress AHRR but with AHRR acting to repress AHR [71]. Thus, AHRR hypomethylation and overexpression in exposed normal cells may not lead to AHR inactivity, consistent with our observations. Instead, the observed gradual inactivation of AHR from dysplasia to LCIS and LSCC suggests that the onset of lung cancer may select for cells for which AHR is inactivated. Given that AHR activation in lung epithelia is associated with an enhanced CD4+ T-cell immune response [53, 54], it is plausible that its observed gradual inactivation in dysplasia, LCIS, and LSCC may lead to an altered immune response which facilitates oncogenesis, although the relation between AHR and inflammatory pathways is also complex and strongly model dependent [54]. To the best of our knowledge, however, the potential role of AHR inactivity in compromising a healthy immune response sheds entirely novel insight into the potential causal role of the AHR pathway in lung carcinogenesis;
4. another interesting TF is HIF3A, which according to our model exhibits gradual inactivation between dysplasia, LCIS, and LSCC (Fig. 5, Table 1). Given that HIF3A is highly expressed in alveolar epithelial cells and thought to protect cells from hypoxia-induced damage [52], it is tempting to speculate that its inactivation may predispose cells to DNA damage, contributing to the onset of lung dysplasia and carcinoma.

Beyond identifying key TFs which may be causally implicated in lung cancer etiology, other contributions of this study include the following. First, we have built and validated a high-confidence regulatory network for lung tissue using two of the largest RNA-seq compendia, encompassing > 30 tissue types and almost 9000 samples. The construction of this network used partial correlations to remove likely indirect associations and further used a strategy to ensure that the TFs overexpressed in lung tissue are not due to immune-cell contamination. Second, using this lung-specific regulatory network, we have shown that it is possible to successfully infer TF activity in independent samples, using either mRNA expression or promoter DNAm patterns. Importantly, using three independent mRNA expression datasets, we have shown that SEPIRA improves the sensitivity to detect lung-specific TFs compared to simple differential expression analysis, in line with previous studies who have shown the feasibility and added value of predicting TF activity from the gene expression values of a high-confidence set of TF targets (see e.g. [33]). In this regard, it is worth pointing out that SEPIRA does not require expression values for the TF of interest in order to infer TF activity and that it also does not require expression values for all predicted targets. As long as expression values are available for a sufficient number of the predicted targets, inference of TF activity is possible. Of particular novel importance is the demonstration that similar inference of TF activity can be achieved by using only promoter DNAm patterns. While we acknowledge that promoter DNAm patterns are only imperfect predictors of gene expression (compared to say histone modifications [72]), the novel strategy used here to infer the downstream targets using co-expression correlations over a very large number of tissue types is likely to hone in on downstream targets (direct or indirect) that are under epigenetic regulation [73]. Future work may attempt to infer TF activity using DNAm patterns for the enhancers linked to the genes identified in LungNet, using enhancer-promoter networks [74, 75]. A third important contribution of our work is the demonstration (further confirming our previous observation [24]) that inactivation of tissue-specific TFs is an event that appears to be under positive selection in the corresponding cancer type. This key observation suggests that a potential subset of these TFs may be causally implicated in the progression to cancer. A novel aspect of this study is that this result was derived using estimates of TF activity, as opposed to TF expression (which was used in our previous work [24]). Consistent with the results obtained on the normal-tissue expression sets, the results in lung cancer and LCIS further point towards TF binding activity (as estimated using SEPIRA) as a better measure of TF activity than gene expression. Fourth, we have extended all of these observations to the demonstration that a substantial number of these TFs already become inactivated in precursor lung cancer lesions (LCIS), further supporting the view that their inactivation is an early event which is under positive selection. Of note, this result was obtained in two separate LCIS cohorts using different data types (mRNA expression and DNAm). Fifth, the algorithm SEPIRA, which was used to construct the tissue-specific regulatory network and estimation of TF binding activity, is of a general nature and could be applied to any tissue type present in the GTEX database. The ability to infer regulatory activity from a DNAm profile further opens up its application to EWAS and cancer epigenome studies, offering a complementary approach to other recent methods [76].

While SEPIRA has led to novel insights into potential mechanisms underlying lung carcinogenesis, there are of course a number of limitations which need to be pointed out. First, although we did adjust for immune-cell infiltration, other stromal infiltrates (e.g. fibroblasts, adipocytes) may explain the presence of some of the TFs in our list. For instance, this is the case of TGBI1I, a marker of smooth muscle cells, which also exhibited inactivation in dysplasia, LCIS, and LSCC (Fig. 5, Table 1). Thus, the observed changes in TGFBI1I activity could be due either to alterations in the stromal milieu within the lung microenvironment or to DNAm alterations in the stromal cells themselves. At present we cannot distinguish between these two possibilities. A similar limitation applies to the patterns of alteration for all other TFs, as these could be due to changes in the epithelial cell composition of the lung or due to selection of specific lung progenitor/stem cells. We envisage that as the full repertoire of cell types within tissues gets mapped at the transcriptome and epigenome levels [77], that improved cell-type deconvolution methods [45, 78–81] will help clarify these outstanding issues. Another potential limitation of our study is that we ignored other regulatory players (e.g. microRNAs [miRNA] [82]), when constructing LungNet. However, it is generally well accepted that TFs play a more prominent role in controlling the larger tissue-specific changes in gene expression (such as in development and reprogramming). Moreover, although inferring miRNA activity from the expression of predicted targets is also possible [83], this has not yet been clearly demonstrated using DNAm patterns. In contrast, DNAm patterns at regulatory elements exhibit a fairly strong and generally inverse association with TF binding [31].

## Conclusions

Using a novel systems-epigenomics algorithm (SEPIRA) for inferring TF binding activity from either gene expression or DNAm data, we have shown that lung-specific TFs become consistently and preferentially inactivated in lung cancer, in precursor lung cancer lesions, and in some instances also in dysplasias and normal cells exposed to smoke carcinogens. Our data point towards inactivation of the AHR pathway and not hypomethylation of the repressor AHRR, as the more fundamental and potentially causal event in smoking-mediated lung carcinogenesis. We therefore anticipate that SEPIRA will be a useful general tool to identify disrupted regulatory networks in a wide range of different studies, including EWAS.

## Methods

### RNA-seq datasets

We used two RNA-seq dataset compendia, one from GTEX (https://www.gtexportal.org/home/) [44] and another one generated as part of the ProteinAtlas project [55] and which is available from the EBI arrayexpress (E-MTAB-2836). The GTEX dataset was used for construction of LungNet, whereas the NormalAtlas set was used for validation. In the case of GTEX, we downloaded the normalized RPKM data for 23,929 unique Entrez gene IDs and 8555 samples. Data were further log-transformed via log_2_(RPKM + 1). The 8555 samples encompassed 30 tissue types, of which 320 were lung. In the case of ProteinAtlas, we downloaded the normalized RPKM RNA-seq data, which was available for 25,020 unique Entrez gene IDs and 200 samples, encompassing 32 tissue types of which eight were lung samples. Data were log-transformed using the transformation log_2_(RPKM/10 + 1). The factor of 10 was introduced to reduce the unrealistic dynamic range for lowly expressed genes (RPKM < 10), as assessed from studying the distribution of RPKM values.

### Other mRNA expression datasets encompassing normal tissue types

Two additional datasets were used for comparing SEPIRA’s sensitivity to detect lung-specific TFs against using simple differential expression analysis. One dataset is from Roth et al., [59] consisting of 21,025 Entrez gene IDs and 353 samples, encompassing 65 different anatomical regions/tissues in the human body, including three from lung tissue, while the other was drawn from Su et al. [60], comprising 13,262 Entrez gene IDs and 158 samples, encompassing 79 human tissues, including four from lung tissue. In all cases, the normalized datasets were downloaded from GEO (GSE1133 and GSE3526). Probes mapping to same Entrez gene IDs were averaged and data further quantile-normalized using the limma package [84]. Differential expression analysis between lung tissue and all other tissues was performed using an empirical Bayes framework as implemented in limma [84, 85].

### Cancer TCGA RNA-seq and Illumina 450 k datasets

We downloaded and processed level-3 Illumina 450 k and RNA-seqV2 data from the TCGA [86], as described by us previously [87]. Here, we specifically focused on LSCC, consisting of 45 NADJ samples and 473 cancers (RNA-seq) and 41 NADJ samples and 275 cancers (Illumina 450 k DNAm). In addition, to assess specificity of TF-activity changes in cancer, we also considered the RNA-seq data of LUAD, the two types of kidney cancer (KIRC/KIRP), colon cancer (COAD), and bladder cancer (BLCA). Data were processed as described by us previously [87].

### Illumina DNAm 450 k set from the Stem-Cell-Matrix Compendium (SCM2)

We processed an Illumina 450 k dataset generated as part of SCM2 [61] and which we have previously analyzed [24]. We used the same normalized data as in our previous publication, consisting of 479,328 probes (after QC) and 153 samples. Here, we only used the 60 samples from somatic tissues, which included seven lung tissue samples and 53 samples from other tissues. In total, there were 11 tissues represented: lung (*n* = 7), adrenal (*n* = 5), blood (*n* = 2), pancreas (*n* = 2), bladder (*n* = 2), heart (*n* = 5), skeletal muscle (*n* = 2), ureter (*n* = 2), spleen (*n* = 5), thymus (*n* = 2), adipose (*n* = 2), stomach (*n* = 6), brain (*n* = 5), liver (*n* = 4), kidney (*n* = 5), tongue (*n* = 2), and lymph node (*n* = 2).

### Gene expression dataset encompassing all major stages in lung carcinogenesis

We downloaded a normalized Agilent (whole human genome microarray 4x44K G4112F) gene expression dataset encompassing 122 samples from a total of 77 patients from GEO under accession number GSE33479 [63]. The samples correspond to all major states: normal (*n* = 27), hyperplasia (*n* = 15), metaplasia (*n* = 15), dysplasia (*n* = 38), LCIS (*n* = 13), and LSCC (*n* = 14).

### DNA methylation data of LCIS

Illumina 450 k DNAm profiles were generated for 56 lung tissue samples, of which 21 were NADJ tissue and 35 were LCIS. This dataset was analyzed by us previously [6]. We used the same probe-level normalized DNAm dataset as in our previous publication. To assign a unique DNAm value to each gene, we used the same procedure as described above for the TCGA dataset.

### EWAS of smoking in buccal tissue

Illumina 450 k DNAm profiles were generated for buccal samples from 790 women, all aged 53 years at sample draw, as described by us previously [6]. Extensive epidemiological information for all 790 women is available. We used SPY as a measure of smoking exposure, as this better approximates lifetime exposure to smoke carcinogens and its effect is also better reflected in DNAm data [6]. We used the normalized probe-level data as used in our previous publication and followed the same procedure as described for the TCGA dataset to assign a unique DNAm value to each gene.

### Construction of LungNet: a lung-specific TF-regulatory network

Here, we describe the construction of LungNet. From the GTEX dataset, we selected genes with a standard deviation (as assessed over the 8555 samples) of at least 0.25, to remove genes of little or no variance. This left a total of 19,478 genes. We then computed PCCs between a total of 1313 human TFs (we used the curated human TF list from MSigDB) and all non-TF genes (a total of 18,165 genes), over all 8555 samples. PCCs were Fisher z-transformed and *P* values of significance estimated using as the null distribution a Normal centered at 0 and with a standard deviation equal to 1/√nT-3 where nT is the number of distinct tissues (nT = 30). We note that although PCCs were estimated over 8555 samples, we used the effective number of samples which is the number of tissues. This was done to impose a more stringent criterion for statistical significance but also to remove the bias due to intrinsic correlations between samples within the same tissue type. As a significance threshold we used the Bonferroni level (0.05/(1313*18165) = 2e-9). From the correlation matrix, we constructed a binary matrix with 1 indicating significant correlation/anti-correlation and 0 indicating no significant association. Thus, a unit entry between a TF and a gene *g*, means that gene *g* is a potential target of the TF. Next, we selected those TFs with at least ten predicted targets, leaving 938 TFs. For each gene potentially regulated by at least two TFs, we then computed partial correlations between all variables (i.e. the gene plus all TFs potentially regulating that gene). We used an absolute partial correlation coefficient threshold of 0.2 to identify the TFs more likely to be regulating the gene. Given that correlations and partial correlations were estimated > 8555 samples, a threshold of 0.2 is extremely conservative. Thus, partial correlations between *g* and TFs < 0.2 were set to zero in the binary gene-target TF matrix. After this filtering step, some TFs may have < 10 gene targets and these were removed, leaving 722 TFs. Finally, we used an empirical Bayes framework (the limma package [84, 85]) to select the subset of TFs more highly expressed in lung tissue compared to: (1) all other tissues (moderated t-test, *P* value < 0.05 and log_2_FC > 1); and (2) only by comparison to blood and spleen (moderated t-test, *P* value < 0.05 and log_2_FC > 1.5).The latter comparison was included since lung tissue exhibits a relatively high level of immune-cell infiltration, hence by demanding that a TF be significantly more highly expressed in lung compared to blood and spleen, we guarantee that we select the TFs identified in (1) which are not immune-cell markers. This resulted in a lung-specific regulatory network (called “LungNet”) consisting of 38 TFs more highly expressed in lung compared to other tissues and a total of 1145 (non-TF) gene targets. In LungNet, there are 1511 regulatory interactions, of which 1438 are positive and 73 are negative. The number of targets per TF in LungNet was in the range of 10–152 and the number of regulators of genes was in the range of 1–5.

### Inferring TF activity using LungNet

Having constructed LungNet, we then estimate activity of a TF *t* in a given sample *s*, by first z-score normalizing the expression profile of each gene *g* in LungNet across all the samples in a given dataset. We then perform a regression of a sample’s gene expression profile against the binding profile of the given TF, i.e. a corresponding vector with + 1 encoding positive regulations, – 1 negative regulations, and 0 no regulation. We interpret the t-statistic of this linear regression as a proxy to the activity level of the TF *t* in the given sample *s.* These TF-activity levels should be interpreted as relative activity levels, to be interpreted in the context of the dataset. Observe that the estimation of activity levels is not done in a multivariate regression as we have already taken into the account multiple regulation in the construction of LungNet itself.

### Validation of LungNet in the NormalAtlas set

We used the above procedure to estimate TF-activity levels of the 38 TFs in each of the 200 samples from the NormalAtlas RNA-seq set and used t-statistics to determine which TFs exhibit higher levels in lung tissue compared to all other tissues. To further test significance, we randomized the targets within LungNet, keeping the number of targets per TF fixed, re-estimated TF-activity levels.

### Inferring TF activity integrating promoter DNAm levels with LungNet

Although promoter DNAm levels are imperfect correlates of gene expression, we posited that relative TF activity could be inferred by regressing the sample’s promoter DNAm profile (defined over the targets defined in LungNet) against the corresponding TF binding profile. To assign a unique DNAm value to each gene from Illumina 450 k/EPIC bead array data, we used a previously validated procedure [88]. This procedure uses the average DNAm over probes mapping to within 200 bp of the TSS. If no such probes are present on the beadarray, we estimate the average using probes mapping to the first exon. If these are also not present for the given gene, we use the average over probes mapping 1500 bp upstream of the TF. The 200-bp region upstream of the TSS, first exon region, and 1500 bp upstream of the TF are the most predictive regions of gene expression, in the context of Illumina beadarray probe representation [88], which justifies the above procedure. Having assigned a unique DNAm value to each gene, we then z-score normalize the DNAm profile of each gene across samples and estimate TF activity as the t-statistic of a linear regression of this z-score normalized DNAm profile against the TF binding profile, reversing the signs of + 1 and – 1 in LungNet, since lower promoter DNAm levels are normally associated with TF binding.

### Enrichment analysis of LungNet targets among binding targets of TFs using ChIP-Atlas data

For 19 TFs in LungNet, we found corresponding ChIP-seq profiles in ChIP-Atlas (http://chip-atlas.org), which contains over 25,000 ChIP-seq profiles from public repositories, including NCBI, DDBJ, ENA, ENCODE, and the Epigenomics Roadmap. For these 19 TFs, we downloaded the predicted binding targets from ChIP-Atlas using ± 1 kb, ± 5 kb, and ± 10 kb windows to assess overlap between ChIP-seq peaks and the TSS of genes. For each TF and window size we used all predicted binding targets with an average binding score larger than 0, as assessed over all available samples/cell lines. For each of the 19 TFs, we computed the overlap of the ChIP-Atlas binding targets and the predicted targets from LungNet, estimating a *P* value of enrichment using a one-tailed Fisher’s exact test. We verified the validity of the *P* values with 10,000 Monte Carlo randomizations whereby for each TF, an equal number of gene targets in LungNet were randomly selected from the full GTEX dataset. *P* values were adjusted for multiple testing using Benjamini–Hochberg procedure.

### Software availability

R-scripts implementing SEPIRA are freely available from http://github.com/aet21/SEPIRA.

## Additional files

Additional file 1: Additional file containing all Additional file 1: Figures S1–S10 and Table S2. (PDF 1142 kb)

Additional file 2: Excel table containing LungNet. (XLS 325 kb)

Additional file 3: Excel table containing the results of a GSEA on the target genes in LungNet. (XLS 242 kb)

## Abbreviations

DNAm: DNA methylation
GSEA: Gene Set Enrichment Analysis
TCGA: The Cancer Genome Atlas
TF: Transcription factor
TSS: Transcription start site.
