# Supplementary material for: Systems-epigenomics inference of transcription factor activity implicates aryl-hydrocarbon-receptor inactivation as a key event in lung cancer development
Source: Genome Biol. 2017 Dec 20;18:236. doi: 10.1186/s13059-017-1366-0 (PMC5738803; doi:10.1186/s13059-017-1366-0)
Supplement: Supplementary file 1 — Additional file containing all Additional file 1: Figures S1–S10 and Table S2. (PDF 1142 kb) [file 13059_2017_1366_MOESM1_ESM.pdf]

Additional Data File 1 for:

Systems-epigenomics inference of transcription factor activity implicates aryl-hydrocarbon-receptor inactivation as a key event in lung cancer development.

Yuting Chen [1,4] , Martin Widschwendter [2] and Andrew E. Teschendorff [1,2,3,4,*]

1. CAS Key Laboratory of Computational Biology, CAS-MPG Partner Institute for Computational Biology, 320 Yue Yang Road, Shanghai 200031, China.
2. Department of Women's Cancer, University College London, 74 Huntley Street, London WC1E 6AU, United Kingdom.
3. Paul O'Gorman Building, UCL Cancer Institute, University College London, 72 Huntley Street, London WC1E 6BT, United Kingdom.
4. Equal Contribution.

*Corresponding author: Andrew E. Teschendorff- a.teschendorff@ucl.ac.uk , andrew@picb.ac.cn

SUPPLEMENTARY FIGURES

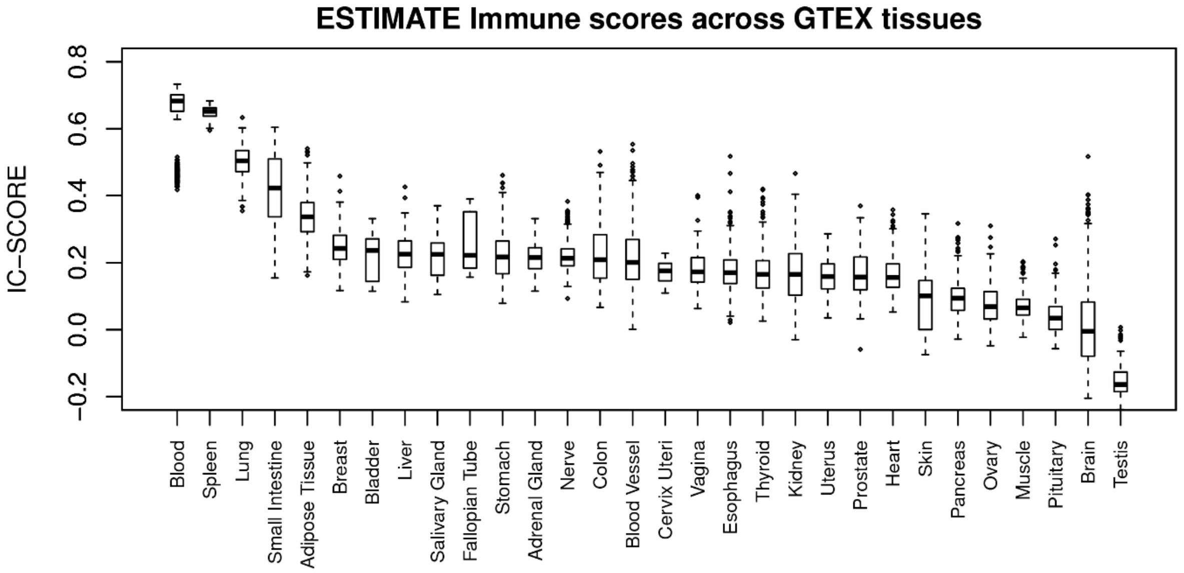

Fig.S1: ESTIMATE immune-cell scores in GTEX dataset. Boxplots of immune-cell infiltrate scores in the GTEX RNA-Seq dataset, stratified according to tissue-type, as obtained using the ESTIMATE algorithm [1]. Tissues have been ranked in decreasing order of immune-cell infiltration. Observed how lung is ranked 3rd, after blood and spleen.

-/+1kb	-/+5kb	-/+10kb	
NA	NA	NA	TFEC
NA	NA	NA	TBX2
0.698	0.326	0.264	FOXA2
0.137	0.077	0.049	TAL1
NA	NA	NA	TBX4
0.777	0.903	0.978	NKX2-1
0.587	0.275	0.019	GATA2
0.137	0.017	0.004	EPAS1
NA	NA	NA	FOXJ1
NA	NA	NA	LDB2
1e-05	4e-05	3e-05	ETS1
0.956	0.926	0.978	ETV1
0.23	0.005	0.016	ERG
0.002	1e-10	2e-11	ELF3
NA	NA	NA	SOX13
0.021	0.005	0.007	AHR
0.053	0.005	0.001	PML
0.052	0.154	0.215	FOXA1
NA	NA	NA	MLLT4
NA	NA	NA	BGN
NA	NA	NA	ZFP36
NA	NA	NA	TNXB
NA	NA	NA	SOX18
NA	NA	NA	TEAD2
1e-05	2e-06	7e-06	XBP1
NA	1	1	MEOX2
0.23	0.013	0.048	KLF4
NA	NA	NA	HIF3A
NA	NA	NA	LSR
NA	NA	NA	KLF9
NA	NA	NA	STON1
1e-04	0.005	0.019	PPARG
NA	NA	NA	ZFP36L2
0.021	0.005	0.001	CEBPD
NA	NA	NA	TRIP10
0.674	0.078	0.049	NR2F2
NA	NA	NA	TGFB1I1
0.364	0.017	0.019	EHF

Fig.S2: Enrichment of ChIP-Seq binding sites among LungNet TF target genes. Heatmap tabulates the enrichment P-values (as derived using a one-tailed Fisher's exact test) for direct binding targets derived from ChIP-Seq profiles (using window sizes of +/- 1kb, 5kb and 10kb, as indicated) of the given TFs, among the corresponding inferred targets using LungNet. For each TF, all available ChIP-Seq profiles for that TF were integrated, irrespective of cell/sample type, as derived from the ChIP-Atlas resource (http://chip-atlas.org). In the table, NA indicates that ChIP-Seq data for that TF was not available, or that not sufficient binding targets were found. In red, we highlight the significant P-values of enrichment, after correction for multiple testing using Benjamini-Hochberg procedure. The P-values are near identical to those obtained using 10,000 Monte-Carlo randomizations whereby for each TF, an equal number of targets in LungNet were randomly selected from the full GTEX dataset.

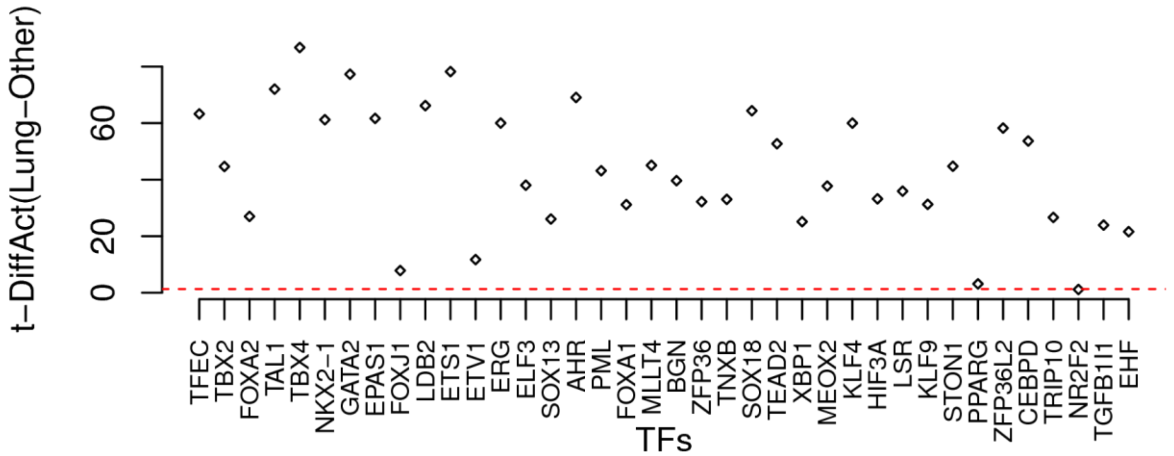

Fig.S3: Verification of estimated TF-activity levels in GTEX. For each of the 38 TFs in LungNet (x-axis), we plot the t-statistics of differential activity between lung and all other tissues (y-axis), as estimated in the GTEX dataset [2]. Red dashed line indicates the line P=0.05. Observe how all but one TF (NR2F2) pass the nominal significance threshold.

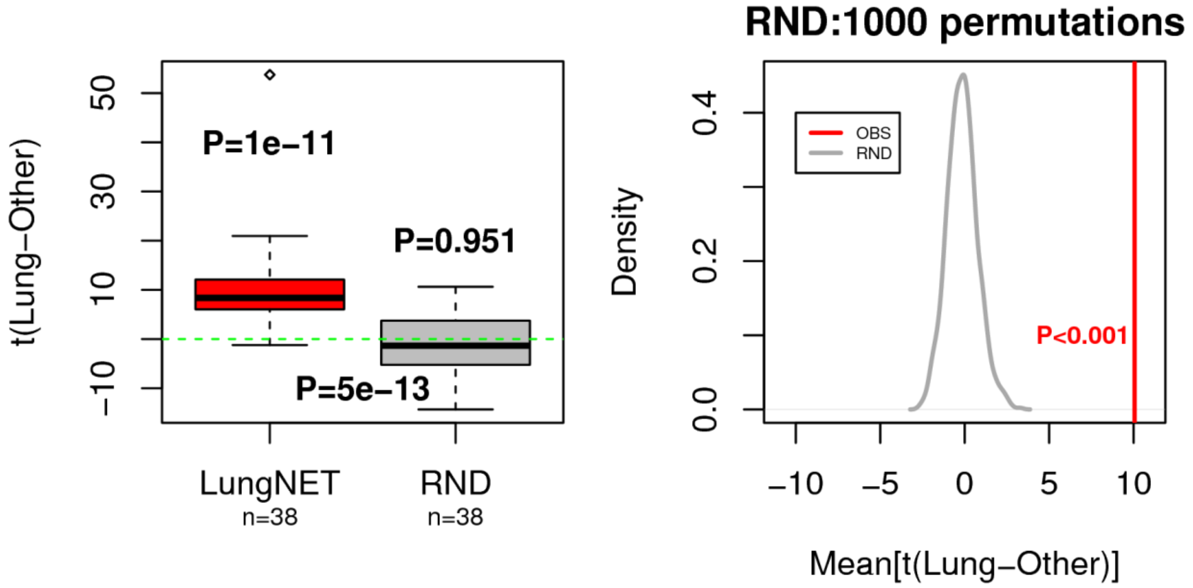

Fig.S4: Monte-Carlo randomization analysis in the NormalAtlas RNA-Seq set [3]. For each of the 38 TFs in LungNet we performed 1000 distinct randomizations, whereby its gene targets were randomized among all possible non-TF genes. For each randomization, we recomputed a t-statistic of differential activity between lung tissue (n=8) and all other tissue types (n=192). Boxplot compares the t-statistics of differential activity for the observed (ie unpermuted case, red) against the average over the 1000 randomizations (grey). P-values are from a Wilcoxon rank sum test: from left to right, the P-value testing that the observed t-statistics are larger than 0, the P-value between the observed (red) and randomized (grey) values, and the P-value for testing that the t-statistics from the randomized case are higher than 0. The density curves in the right panel compare the observed average value over the 38 TFs (red line) to the distribution of the average over the 1000 different randomizations. No randomization led to an average t-statistic larger than the observed one (P<0.001).

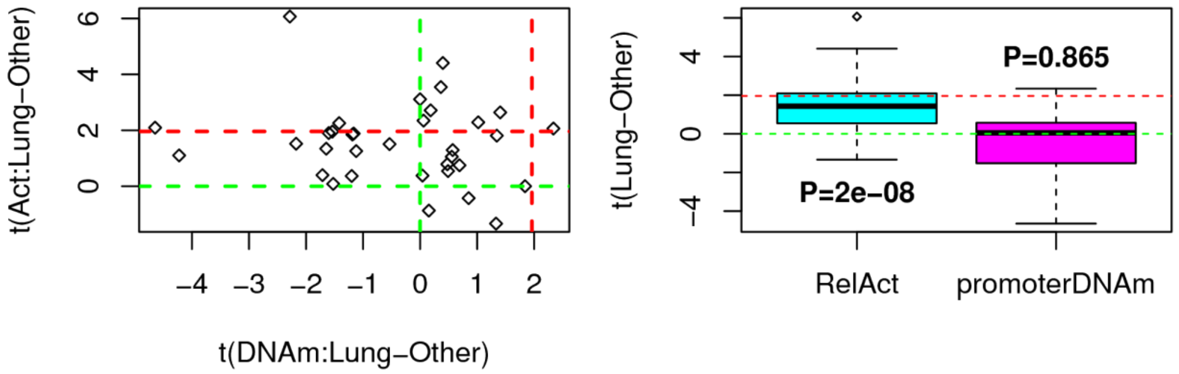

Fig.S5: Comparison of differential activity estimated using SEPIRA vs promoter DNAm in the SCM2 dataset. Left panel: scatterplot of t-statistics of differential activity between lung tissue and all other 10 tissues in the SCM2 Illumina 450k DNAm set [4], for the 38 TFs in LungNet with TF-activity estimated using the SEPIRA algorithm (y-axis) against the corresponding t-statistics of the same 38 TFs estimated from comparing promoter DNAm levels between lung and all other tissues. Right panel: boxplot depiction of the left-panel. P-values are from a one-tailed Wilcoxon rank sum test, assessing whether the distribution of t-statistics is significantly larger than 0. The red dashed lines are the lines of significance P=0.05.

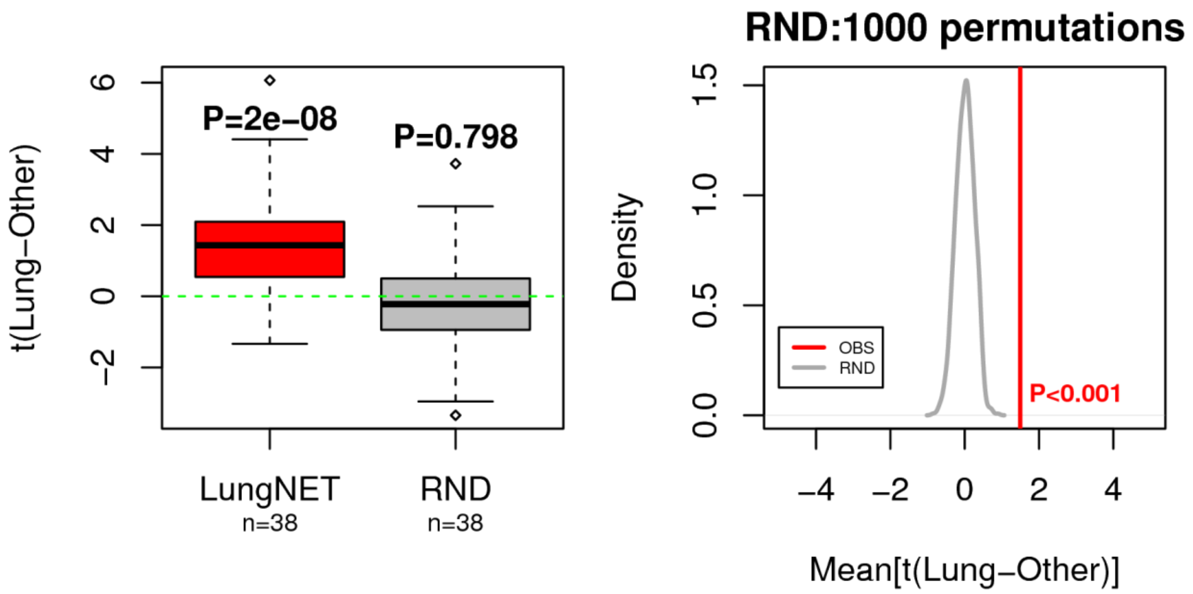

Fig.S6: Monte-Carlo randomization analysis in the SCM2 Illumina DNAm 450k set. For each of the 38 TFs in LungNet we performed 1000 distinct randomizations, whereby its gene targets were randomized among all possible non-TF genes. For each randomization, we recomputed a t-statistic of differential activity between lung tissue (n=7) and all other tissue types (n=53) in the SCM2 Illumina 450k set. Boxplot compares the t-statistics of differential activity for the observed (ie unpermuted case, red) against the average over the 1000 randomizations (grey). P-values are from a Wilcoxon rank sum test: from left to right, the P-value testing that the observed t-statistics are larger than 0, and the P-value for testing that

the t-statistics from the randomized case are higher than 0. The density curves in the right panel compare the observed average value over the 38 TFs (red line) to the distribution of the average over the 1000 different randomizations. No randomization led to an average t-statistic larger than the observed one (P<0.001).

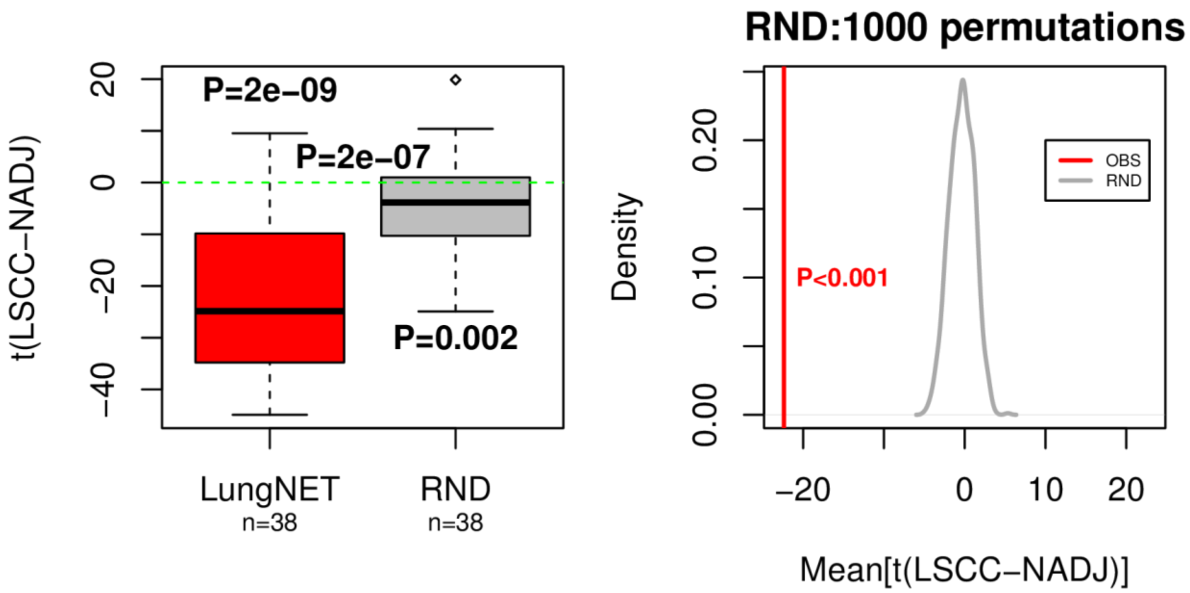

Fig.S7: Monte-Carlo randomization analysis in the TCGA LSCC RNA-Seq set. For each of the 38 TFs in LungNet we performed 1000 distinct randomizations, whereby its gene targets were randomized among all possible non-TF genes. For each randomization, we recomputed a t-statistic of differential activity between LSCC and normal-adjacent tissue from the TCGA LSCC RNA-Seq set [5]. Boxplot compares the t-statistics of differential activity for the observed (ie unpermuted case, red) against the average over the 1000 randomizations (grey). P-values are from a Wilcoxon rank sum test: from left to right, the P-value testing that the observed t-statistics are lower than 0, the P-value testing that the observed t-statistics are lower than the ones for the permuted case, and the P-value for testing that the t-statistics from the randomized case are lower than 0. The density curves in the right panel compare the observed average value over the 38 TFs (red line) to the distribution of the average over the 1000 different randomizations. No randomization led to an average t-statistic lower than the observed one (P<0.001).

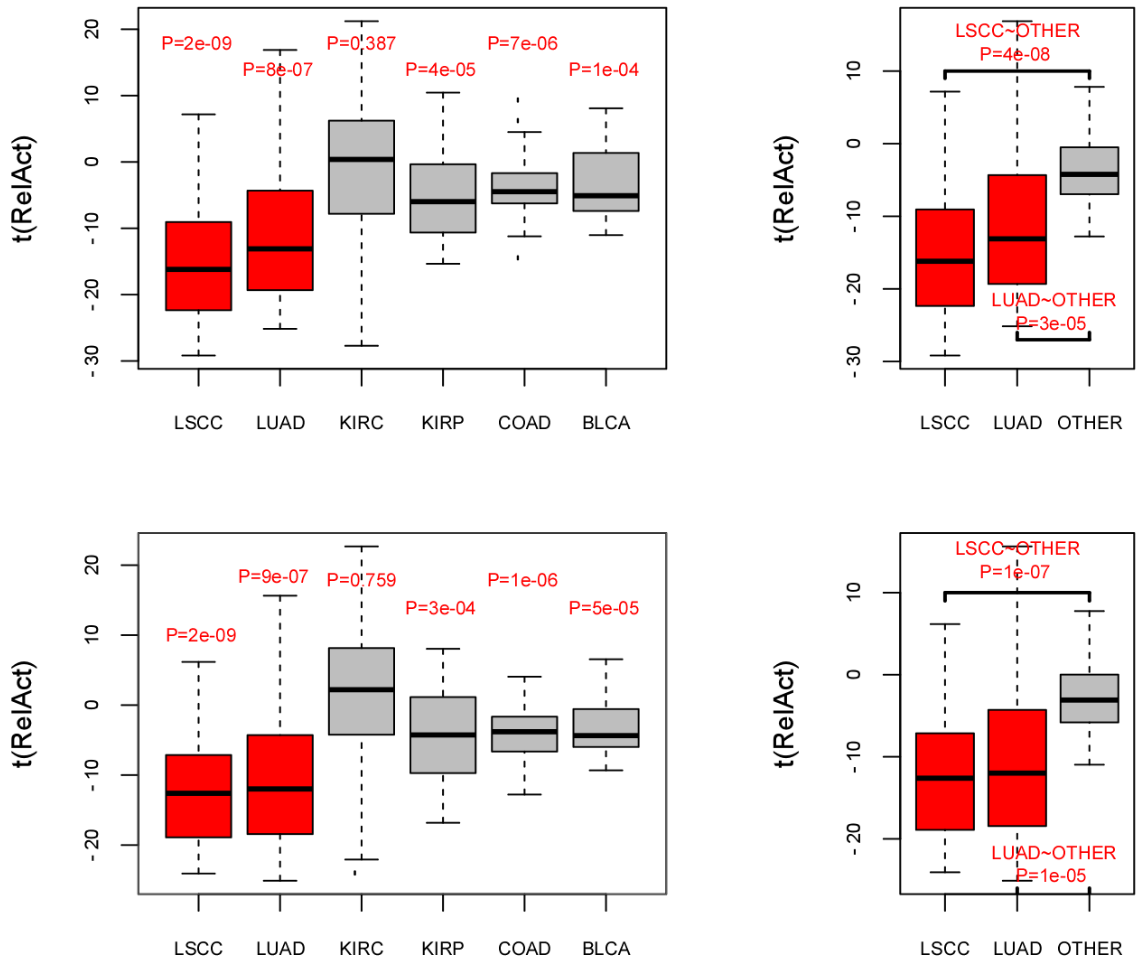

Fig.S8: Preferential inactivation of lung-specific TFs in LSCC and LUAD. Left panels: Boxplots of t-statistics of differential TF binding activity between cancer and normal-adjacent tissue for 6 different TCGA cancer types. TF binding activity was estimated using SEPIRA on the RNA-Seq data. P-values are from a one-tailed Wilcoxon rank sum test. **Right panels:** As left panel but now combining the non-lung cancer types together in one group, and P-values reflecting whether the t-statistics of differential activity are lower in the lung cancers compared to all other cancer types. Top row is for the case where SEPIRA was applied to the RNA-Seq data without prior z-score normalization. Bottom row for the case where z-score normalization was performed on the RNA-Seq data before applying SEPIRA.

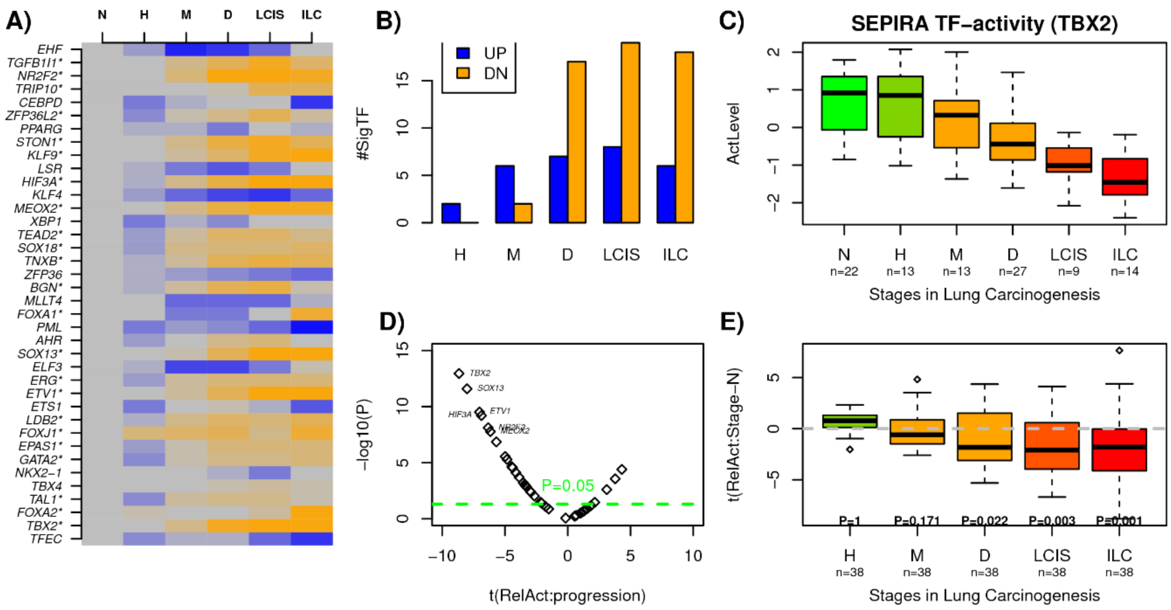

Fig.S9: SEPIRA+LungNet predicts preferential inactivation of lung-specific TFs during progression to lung squamous cell carcinoma, including LCIS. (As Figure-5 but with multiple biopsies per disease stage and patient averaged before estimating TF-activity). A) Heatmap of t-statistics of differential TF activity, as estimated using SEPIRA from a gene expression data matrix encompassing all major histological stages of lung carcinogenesis (N=normal, H=hyperplasia, M=metaplasia, D=dysplasia, LCIS=lung carcinoma in situ, ILC=invasive lung cancer (squamous)). *indicates TFs with significant changes in TF-activity during disease progression. Blue=relative high activity, Orange=relative low activity. **B)** Numbers of significantly deactivated (DN) and activated (UP) TFs in each disease stage relative to normal. **C)** Boxplot of estimated TF-activity levels for TBX2 against disease stage. **D)** Scatterplot of t-statistics from a regression of TF-activity against disease stage (x-axis) against their significance level (-$\log_{10}$P, y-axis). **E)** Boxplots of the t-statistics of differential activity between each disease stage and normal lung. P-values are from a one-tailed Wilcoxon rank sum test, testing that the distribution of the differential activity values is less than 0.

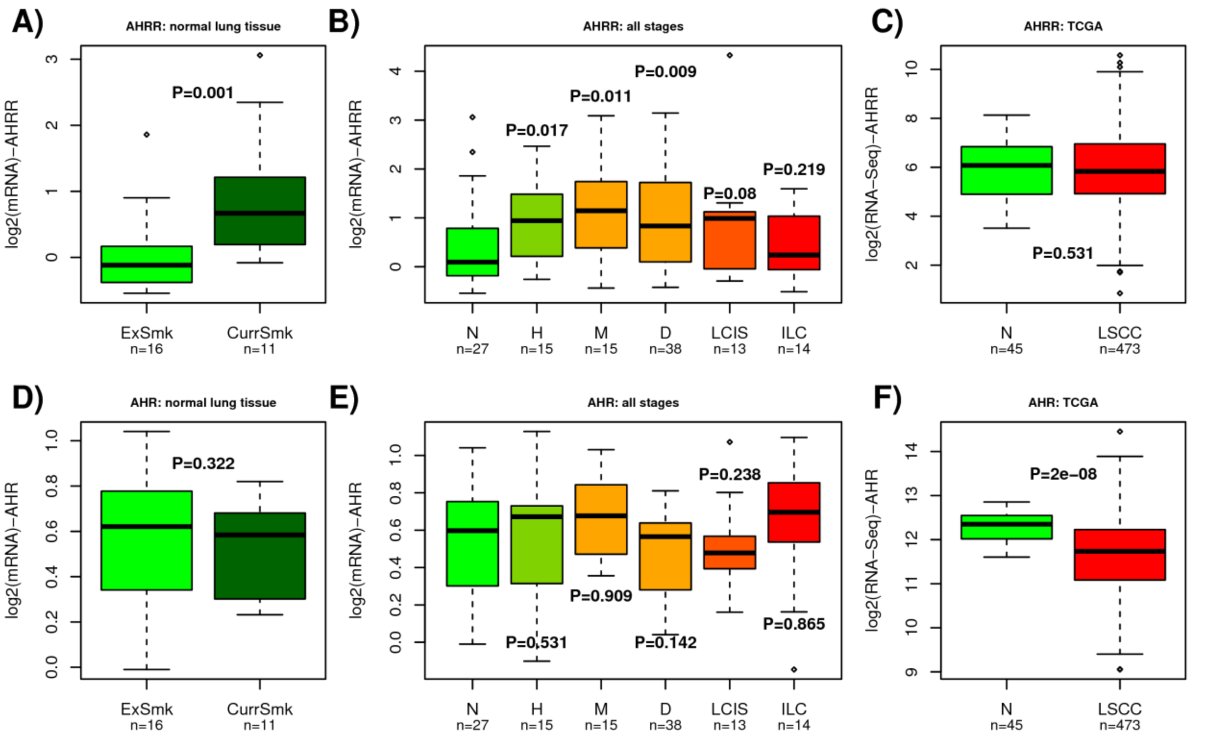

Fig.S10: Contrasting dynamics of differential expression of AHRR and AHR in lung cancer. A) Boxplot of mRNA expression of AHRR in normal lung tissue of ex-smokers and current smokers. P-value is from a one-tailed Wilcoxon rank sum test. **B)** Boxplots of mRNA expression of AHRR across all major histological stages of lung carcinogenesis (N=normal, H=hyperplasia, M=metaplasia, D=dysplasia, LCIS=lung carcinoma in-situ, ILC=invasive lung cancer). P-values are derived from a one-tailed Wilcoxon rank sum test comparing each stage to normal lung (N). **C)** Boxplots of mRNA expression of AHRR between normal-adjacent and lung squamous cell carcinoma samples of the TCGA. P-value is from a one-tailed Wilcoxon rank sum test. **D-F)** As A-C), but now for AHR, the TF which is a target of AHRR.

SUPPLEMENTARY TABLES

Symbol	Entrez ID	nTGTS	Act	Rep
TFEC	22797	33	33	0
TBX2	6909	18	14	4
FOXA2	3170	15	15	0
TAL1	6886	19	19	0
TBX4	9496	16	16	0
NKX2-1	7080	24	24	0
GATA2	2624	13	13	0
EPAS1	2034	85	83	2
FOXJ1	2302	152	152	0
LDB2	9079	63	63	0
ETS1	2113	35	35	0
ETV1	2115	11	11	0
ERG	2078	44	44	0
ELF3	1999	71	71	0
SOX13	9580	14	14	0
AHR	196	39	38	1
PML	5371	33	28	5
FOXA1	3169	10	10	0
MLLT4	4301	26	26	0
BGN	633	93	93	0
ZFP36	7538	19	18	1
TNXB	7148	40	40	0
SOX18	54345	60	60	0
TEAD2	8463	53	52	1
XBP1	7494	18	18	0
MEOX2	4223	42	41	1
KLF4	9314	20	20	0
HIF3A	64344	10	10	0
LSR	51599	70	70	0
KLF9	687	15	15	0
STON1	11037	31	30	1
PPARG	5468	16	16	0
ZFP36L2	678	24	24	0
CEBPD	1052	17	17	0
TRIP10	9322	42	23	19
NR2F2	7026	31	24	7
TGFB1I1	7041	112	108	4
EHF	26298	77	50	27

Table.S1: Properties of LungNet. Table lists the 38 transcription factors in LungNet, plus their number of targets, and their distribution in terms of activating or repressive interactions.

REFERENCES

1. Yoshihara K, Shahmoradgoli M, Martinez E, Vegesna R, Kim H, Torres-Garcia W, Trevino V, Shen H, Laird PW, Levine DA, et al: **Inferring tumour purity and stromal and immune cell admixture from expression data.** *Nat Commun* 2013, **4:**2612.
2. Consortium GT: **The Genotype-Tissue Expression (GTEx) project.** *Nat Genet* 2013, **45:**580-585.
3. Uhlen M, Fagerberg L, Hallstrom BM, Lindskog C, Oksvold P, Mardinoglu A, Sivertsson A, Kampf C, Sjostedt E, Asplund A, et al: **Proteomics. Tissue-based map of the human proteome.** *Science* 2015, **347:**1260419.
4. Nazor KL, Altun G, Lynch C, Tran H, Harness JV, Slavin I, Garitaonandia I, Muller FJ, Wang YC, Boscolo FS, et al: **Recurrent variations in DNA methylation in human pluripotent stem cells and their differentiated derivatives.** *Cell Stem Cell* 2012, **10:**620-634.
5. Cancer Genome Atlas Research N: **Comprehensive genomic characterization of squamous cell lung cancers.** *Nature* 2012, **489:**519-525.
